# Influenza vaccination is associated with a reduced risk of invasive aspergillosis in high-risk individuals in Taiwan: a population-based cohort study

**DOI:** 10.1080/22221751.2022.2155584

**Published:** 2022-12-27

**Authors:** Yi-Jyun Chen, I-Feng Lin, Jen-Hsiang Chuang, Hung-Ling Huang, Ta-Chien Chan

**Affiliations:** a Institute of Public Health, College of Medicine, National Yang Ming Chiao Tung University, Taipei, Taiwan; b Centers for Disease Control, Taipei, Taiwan; c Department of Internal Medicine, Kaohsiung Municipal Ta-Tung Hospital, Kaohsiung Medical University Hospital, Kaohsiung, Taiwan; d Division of Pulmonary and Critical Care Medicine, Kaohsiung Medical University Hospital, Kaohsiung, Taiwan; e Department of Internal Medicine, Kaohsiung Medical University Hospital, Kaohsiung, Taiwan; f Graduate Institute of Medicine, College of Medicine, Kaohsiung Medical University, Kaohsiung, Taiwan; g Research Center for Humanities and Social Sciences, Academia Sinica, Taipei, Taiwan

**Keywords:** Influenza vaccination, influenza superinfection, invasive aspergillosis, chronic diseases, health insurance database

## Abstract

Invasive aspergillosis (IA) has become the emerging life-threatening disease in recent years. Influenza has been identified as an independent risk factor for IA. Vaccination is the most effective way to prevent influenza, while whether it can reduce IA in high-risk population still uncertain. We aimed to investigate the association between influenza vaccination and the risk of IA in high-risk population. We performed a population-based cohort study of people who qualified for government-funded influenza vaccination and were at high risk for IA at the start of the influenza season each year between 2016 and 2019. We utilized Taiwan’s National Health Insurance Research Database to identify the influenza vaccination status and IA diagnosis during the follow-up period. We compared the risk of IA between people with and without vaccination using multivariable logistic regression analysis. Out of total 8,544,451 people who were eligible during the 3 influenza seasons, 3,136,477 (36.7%) were vaccinated. A total of 1179 IA cases with the incidence of 13.8 cases per 100,000 high-risk individuals were identified during the follow-up. Compared to non-vaccinated group, vaccinated individuals had a 21% risk reduction of IA (adjusted odds ratio 0.79, 95% confidence interval 0.70–0.90). Influenza vaccination was associated with a lower risk of IA among males, immunosuppressive conditions, malignancy, diabetes, and those having host factors according to the European Organization for Research and Treatment of Cancer and the Mycoses Study Group Education and Research Consortium. Influenza vaccination is recommended for high-risk population to reduce the risk of IA.

## Introduction

Invasive aspergillosis (IA) is recognized as a life-threatening disease affecting immunocompromised patients, including patients with prolonged neutropenia, inherited immune dysfunction disorders, recipients of allogeneic stem cell or solid organ transplantation who develop severe graft-versus-host disease, and under immunosuppressive therapy [1]. The link between influenza and IA has been reported in recent decades [2–4] and poses as a potentially lethal complication of influenza in critically ill patients with 90-day mortality up to 50% [5]. The demographic variation has been noticed in the incidence of influenza-associated aspergillosis (IAA), with higher rates in Europe and Asia (ranging from 12% to 28%) but lower in North America [6], which might be contributed from patients’ underlying conditions, environmental exposure of *Aspergillus*, available diagnostic tools, different influenza treatment and influenza vaccination policy [7]. IAA has become an important clinical issue as affecting 16.9% of patients with influenza in Taiwan [8].

Annual vaccination can either prevent influenza for higher risk population including elders, children, people with comorbidities, and pregnant women [9], but also reduce influenza-associated complications, with lowering risk of hospitalization, severity of illness, cardiovascular events, respiratory tract superinfection, and all-cause mortality [10–12]. Taiwan has launched a Government-Funded Influenza Vaccination Program for population at high-risk for influenza complications receiving free vaccines since 1998 [13]. Influenza vaccination might reduce the risk of IA by preventing influenza and reducing its severity. However, there is little evidence of an association between influenza vaccination and IA. This population-based cohort study was designed to investigate the association between influenza vaccination and the risk of IA in high-risk population.

## Methods

### Data and study design

We utilized Taiwan’s National Health Insurance Research Database (NHIRD, 2014–2019), which stored the claim data of Taiwan’s National Health Insurance (NHI) that covers about 99.9% of the population [14], to obtain information about demographics, comorbidities, medications, and health care. The Statistics of Communicable Diseases and Surveillance Report from the Taiwan Centers for Disease Control (TCDC) was used to realize the influenza epidemic [15]. The government-funded influenza vaccine is provided to those with HIV infection, immunodeficiency disorders, diabetes, cardiovascular disease, chronic respiratory disorders, chronic liver diseases, autoimmune diseases, chronic kidney disorders, neurological disorders, amyloidosis, asplenia, or obesity; those certificated with catastrophic illness; and those with rare types of diseases [13]. The Government-Funded Influenza Vaccination Program offers reimbursement of the public vaccine injection fee, and health care providers can claim it through the NHI system [13]. The rules of claiming the vaccination fee were rather restricted before the 2016–2017 influenza season, which may affect the validity of vaccination records in the NHIRD. Hence, we only followed three influenza seasons between 2016 and 2019.

As IA is associated with influenza, we only included IA cases from the start of the influenza season till 60 days after the end of the season or until the date before the next season’s public vaccination programme. We defined the influenza season threshold with a 10% influenza culture-positive rate [16]. In each season, we independently enrolled people qualified for government-funded influenza vaccinations and at high-risk for IA to determine whether they were vaccinated or contracted IA. We then pooled data from the three seasons to assess the association between vaccination and IA. This study was approved by the Institutional Review Board of Biomedical Science Research, Academia Sinica (application no: AS-IRB01-21003).

### Study population

Individuals who met the following criteria were enrolled in each season: (1) individuals qualified to receive public influenza vaccine, which was defined as an individual with any of the following: aged 50 years and older, having catastrophic illness (Supplementary Data), and having high-risk comorbidities for influenza complications listed in the vaccination programme (Supplementary Table 1); (2) individuals at high-risk for IA, which was defined as an individual having any of the following conditions: transplantation, malignancy, autoimmune diseases, immunodeficiency, asthma, chronic obstructive pulmonary disease (COPD), other chronic respiratory diseases, chronic kidney disease (CKD), aplastic anaemia, myelodysplastic syndrome, diabetes, liver cirrhosis/failure, heart failure, use of immunosuppressive agents, or using a least 44.1 cumulative Defined Daily Dose (cDDD) of systemic corticosteroids [1,17,18]. People were excluded if the data records lacked sex or residence information, were <20 years old, and died before the season started, were not diagnosed with IA but using antifungal agents, or had been diagnosed with IA before the season began. Individuals were ineligible if their dates of hospitalization for IA were within less than two weeks after vaccination.

### Influenza vaccination

Between 2016 and 2019, the vaccination programme provided trivalent inactive vaccines (TIV). In each season, influenza vaccination was defined as the person’s first vaccination record from the start of the vaccination programme until the end of follow-up. It was defined by the NHI Drug Codes for the influenza vaccine and the code for claiming the vaccine injection fee (Supplementary Table 3).

### IA

IA was defined as the first hospitalization with aspergillosis (International Classification of Diseases, Ninth Revision [ICD-9] codes 117.3, 484.6; ICD-10 codes B44) during the follow-up period and without any IA diagnosis before the influenza season [19]. IA-associated death was defined as individuals with IA that died within 60 and 90 days after hospitalization due to aspergillosis.

### Covariates

We collected data on four categories of covariates: demographics, comorbidities, medications, and healthcare usage. The demographics included age, sex, residential region, and insurance amount in the month of the influenza season. Comorbidities were measured by ICD-10 codes and by the number of outpatient department (OPD) visits and hospitalizations in the 270-day period before the season. ICD codes are listed in Supplementary Table 2. Medications included the use of immunosuppressive agents such as antineoplastic agents and immunosuppressants, whether a person inhaled corticosteroids, and the cDDD of systemic corticosteroids used by 90-day, before the season. Medications were defined using the ATC codes in Supplementary Table 3. Health care use included the number of OPD visits, number of hospitalizations, number of OPD visits for influenza-like illnesses (ILI), and number of hospitalizations with ILI by 270-day before the season started. The ICD codes defining ILI were modified from the codes used by the TCDC in the real-time outbreak and disease surveillance (RODS) system and are listed in Supplementary Table 2 [20].

### Statistical analyses

After independently enrolling eligible people in each season, we combined data from the three seasons. Continuous variables of vaccinated and unvaccinated individuals were compared using the Independent *t*-test or the Mann–Whitney *U* test, as appropriate. Categorical variables were compared using the *χ*^2^ test. Multivariable logistic regression assessed the association between influenza vaccination and IA. As the demographics, comorbidities, medications, and healthcare usage were associated with influenza vaccination and the risk for IA, these factors may potentially confound the association between vaccination and IA. We included them as covariates and described the association by adjusted odds ratio (aOR) and 95% confidence intervals (95% CI).

We tested the association in each season and stratified the patients according to age, sex, approximate host factors defined by the European Organization for Research and Treatment of Cancer and the Mycoses Study Group Education and Research Consortium (EORTC/MSGERC), and comorbidities. The approximate EORTC/MSGERC host factors were hematological malignancy, hematopoietic stem cell transplantation (HSCT), solid-organ transplantation (SOT), immunodeficiency disease, using immunosuppressive agents, and using 44.1 cDDD systemic corticosteroids or above [1]. Comorbidities were classified as those having an immunocompromised status, including transplantation, HIV infection, immunodeficiency disease, using immunosuppressive agents, and using 44.1 cDDD systemic corticosteroids or greater; those having malignancies, including hematological and solid-organ malignancy and metastasis; those with chronic respiratory disease, including asthma, COPD, and other chronic respiratory diseases; and those diagnosed with diabetes with and without chronic complications.

We designed five types of sensitivity analyses: changing the IA definition, shortening the follow-up time, using positive control outcomes, using a negative control outcome, and assessing the association between hospitalization with influenza and IA. The details of the sensitivity analyses are described in the Supplementary Data. All analyses were tested with a two-tailed significance level of 0.05 using SAS version 9.4 (SAS Institute, Cary NC).

## Results

**Figure 1.**
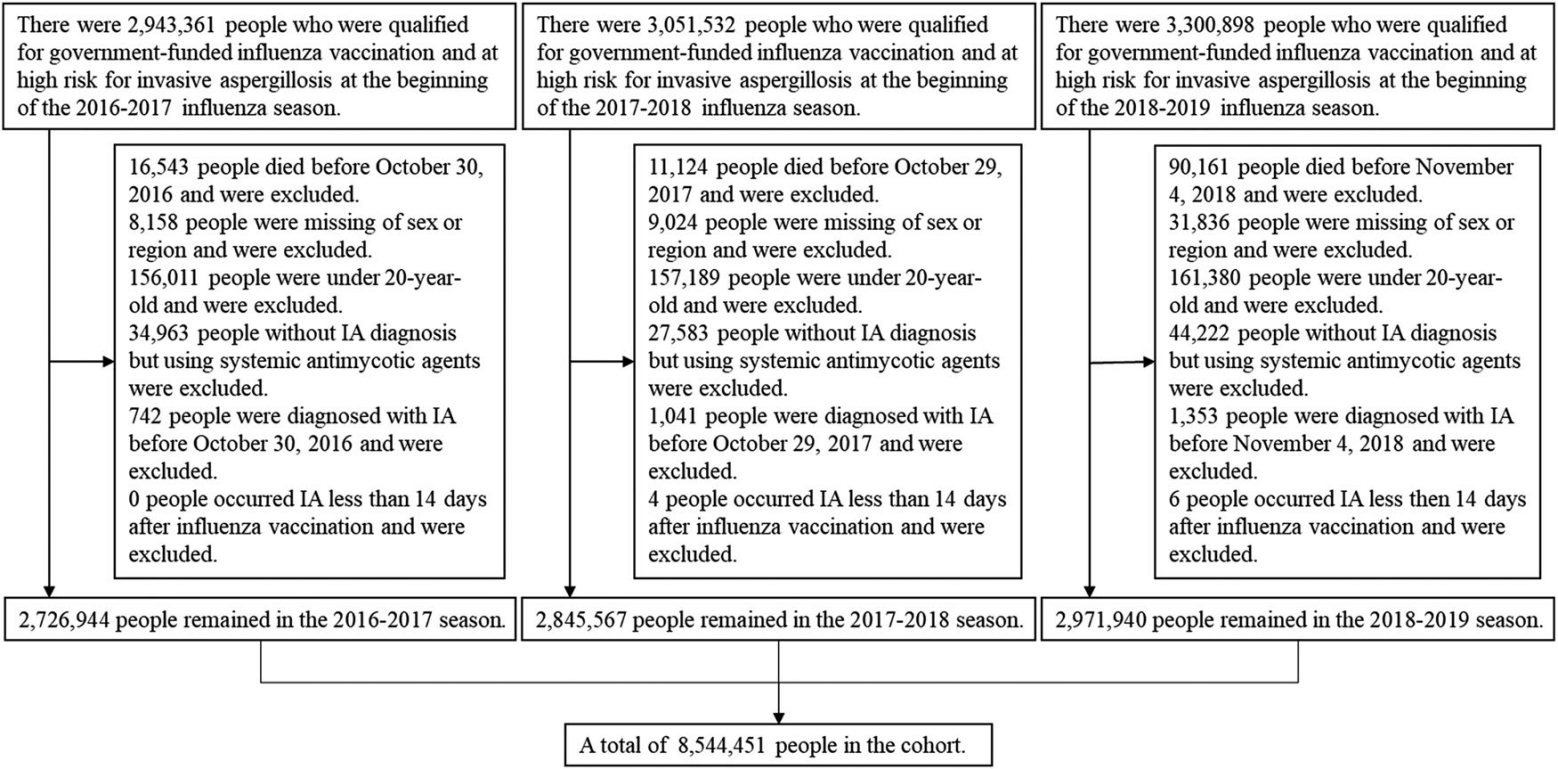
Flow chart of the study population from 2016–2017 to 2018–2019 influenza season. Note: Figure 1 shows how the study population was selected according to the inclusion and exclusion criteria.

Between the 2016–2017 and 2018–2019 influenza season, we included 2,726,944; 2,845,567; and 2,971,940 people, respectively, who were qualified for government-funded influenza vaccination and were at high risk for IA (Figure 1). Of the 8,544,451 people in this study, there were 3,916,287 unique individuals, 722,914 (18.5%) were eligible for 2 seasons, and 1,952,625 (49.9%) were eligible for all 3 seasons.

### Baseline characteristics by vaccination status

The overall baseline characteristics of the patients are listed in Table 1. The mean age of the study population was 63.0 years (standard deviation, 14.1 years) and 4,247,235 people (49.7%) were males. The overall vaccination rate was 36.7%, and the coverage rates ranged from 35.0% to 38.1% for each influenza season. 49.7% of people aged 65 years and older received influenza vaccination, whereas only 25.4% of adults <65 years were vaccinated. Compared with unvaccinated individuals, vaccinated ones tended to be older, had a lower proportion of living in the northern region, had lower insurance amounts, were diagnosed with COPD, and had more OPD visits (Table 1).

**Table 1.** Baseline characteristics of people at high-risk for invasive aspergillosis based on vaccination status.

	Total	Non-vaccinated	Vaccinated	*p*-Value
	*N* = 8,544,451	*N* = 5,407,974	*N* = 3,136,477	36.7%
Age, year, mean (SD)	63.0 (14.1)	60 (14.2)	68.1 (12.4)	<.001
Male sex, *n* (%)	4,247,235 (49.7)	2,709,313 (50.1)	1,537,922 (49)	<.001
Insurance amount, TWD, *n* (%)				<.001
0–20,000	1,963,350 (23)	1,192,316 (22.1)	771,034 (24.6)	
20,000–30,000	3,742,825 (43.8)	2,310,061 (42.7)	1,432,764 (45.7)	
30,000–40,000	931,638 (10.9)	630,480 (11.7)	301,158 (9.6)	
40,000–50,000	899,379 (10.5)	610,331 (11.3)	289,048 (9.2)	
>50,000	1,007,259 (11.8)	664,786 (12.3)	342,473 (10.9)	
Region, *n* (%)				<.001
Northern	3,992,788 (46.7)	2,675,198 (49.5)	1,317,590 (42)	
Central	2,011,773 (23.5)	1,204,337 (22.3)	807,436 (25.7)	
Southern	2,261,372 (26.5)	1,360,602 (25.2)	900,770 (28.7)	
Eastern and offshore islands	278,518 (3.3)	167,837 (3.1)	110,681 (3.5)	
Medications				
Systemic corticosteroids, cDDD, mean (SD)	4.4 (24.2)	4.7 (23.3)	3.8 (25.5)	<.001
Inhaled corticosteroids, *n* (%)	347,818 (4.1)	193,620 (3.6)	154,198 (4.9)	<.001
Immunosuppressive agents, *n* (%)	434,302 (5.1)	317,662 (5.9)	116,640 (3.7)	<.001
Comorbidities, *n* (%)				
Hematopoietic stem cell transplantation	3621 (<0.1)	2467 (0.1)	1154 (<0.1)	<.001
Solid-organ transplantation	34,415 (0.4)	26,564 (0.5)	7851 (0.3)	<.001
Hematological malignancy	73,326 (0.9)	48,778 (0.9)	24,548 (0.8)	<.001
Metastatic malignancy	153,207 (1.8)	118,014 (2.2)	35,193 (1.1)	<.001
Solid-organ malignancy	1,365,984 (16.0)	872,238 (16.1)	493,746 (15.7)	<.001
Autoimmune diseases	536,314 (6.3)	353,565 (6.5)	182,749 (5.8)	<.001
Immunodeficiency disorders and HIV	67,681 (0.8)	37,879 (0.7)	29,802 (1)	<.001
Asthma	793,445 (9.3)	507,048 (9.4)	286,397 (9.1)	<.001
COPD	807,629 (9.5)	416,553 (7.7)	391,076 (12.5)	<.001
Other chronic respiratory diseases	250,708 (2.9)	140,143 (2.6)	110,565 (3.5)	<.001
CKD stage 5 and ESRD	365,049 (4.3)	197,010 (3.6)	168,039 (5.4)	<.001
CKD stage 1–4	123,279 (1.4)	71,225 (1.3)	52,054 (1.7)	<.001
Aplastic anaemia	12,077 (0.1)	8286 (0.2)	3791 (0.1)	<.001
Myelodysplastic syndrome	6084 (0.1)	3791 (0.1)	2293 (0.1)	.112
Diabetes with chronic complications	1,339,825 (15.7)	784,058 (14.5)	555,767 (17.7)	<.001
Diabetes without chronic complications	3,619,027 (42.4)	2,293,585 (42.4)	1,325,442 (42.3)	<.001
Liver cirrhosis/failure	266,968 (3.1)	182,772 (3.4)	84,196 (2.7)	<.001
Heart failure	666,305 (7.8)	392,398 (7.3)	273,907 (8.7)	<.001
Health care use, mean (SD)				
No. of admissions	0.4 (1)	0.4 (1.1)	0.3 (0.9)	<.001
No. of OPD visits	17.2 (12.3)	15.3 (11.3)	20.6 (13.1)	<.001
No. of admissions for ILI	0.1 (0.4)	0.1 (0.4)	0.1 (0.4)	<.001
No. of OPD visits for ILI	3 (4.7)	2.7 (4.3)	3.5 (5.1)	<.001

Abbreviations: SD, standard deviation; cDDD, cumulative defined daily dose; HIV, human immunodeficiency virus; COPD, chronic obstructive pulmonary disease; CKD, chronic kidney disease; ESRD, end-stage renal disease; No., number; OPD, outpatient department; ILI, influenza-like illness.

### Association between influenza vaccination and IA

A total of 1179 IA cases were diagnosed during the 3 influenza seasons, with an incidence of 13.8 cases per 100,000 high-risk individuals. The results of this analysis are summarized in Table 2. Before adjustment, vaccination was not associated with IA (crude odds ratio 0.93, 95% CI 0.82–1.04). After adjusting for covariates, vaccination was associated with a significantly reduced risk of IA (aOR 0.79, 95% CI 0.70–0.90). However, vaccinated individuals did not have a significantly lower IA-associated mortality (Table 3).

**Table 2.** Association between influenza vaccination and invasive aspergillosis.

Outcome	IA/vaccinated	IA/non-vaccinated	cOR (95% CI)	aOR^a^ (95% CI)
Overall	412/3,136,477	767/5,407,974	0.93 (0.82–1.04)	0.79 (0.70–0.90)
Age				
≥65	312/1,975,560	362/1,997,288	0.87 (0.75–1.01)	0.85 (0.73–0.99)
<65	100/1,160,917	405/3,410,686	0.73 (0.58–0.90)	0.63 (0.50–0.79)
Sex				
Male	264/1,537,922	494/2,709,313	0.94 (0.81–1.09)	0.78 (0.67–0.92)
Female	148/1,598,555	273/2,698,661	0.92 (0.75–1.12)	0.82 (0.67–1.02)
EORTC/MSGERC host factors^b^				
With host factors	126/198,100	334/460,964	0.88 (0.72–1.08)	0.78 (0.63–0.97)
Without host factors	286/2,938,377	433/4,947,010	1.11 (0.96–1.29)	0.79 (0.67–0.92)
Comorbidities				
Immunocompromised status	113/208,690	313/463,639	0.80 (0.65–1.00)	0.76 (0.61–0.95)
Malignancy	129/549,610	306/1,031,442	0.79 (0.64–0.97)	0.80 (0.64–0.99)
Chronic respiratory diseases	196/669,448	239/943,528	1.16 (0.96–1.40)	0.98 (0.81–1.20)
Diabetes	174/1,881,209	286/3,077,643	1.00 (0.82–1.20)	0.76 (0.63–0.93)
Season				
2016–2017	124/1,012,935	226/1,714,009	0.93 (0.75–1.16)	0.85 (0.67–1.07)
2017–2018	118/1,082,845	186/1,762,722	1.03 (0.82–1.30)	0.90 (0.71–1.15)
2018–2019	170/1,040,697	355/1,931,243	0.89 (0.74–1.07)	0.72 (0.59–0.87)

Abbreviations: IA, invasive aspergillosis; cOR, crude odds ratio; aOR, adjusted odds ratio; EORTC/MSGERC, European Organization for Research and Treatment of Cancer and the Mycoses Study Group Education and Research Consortium.

^a^ Adjusting for age, sex, insurance amount, region, systemic corticosteroids, inhaled corticosteroids, immunosuppressive agents, hematopoietic stem cell transplant, solid-organ transplantation, hematological malignancy, metastatic malignancy, solid-organ malignancy, autoimmune diseases, immunodeficiency disorders, HIV, asthma, COPD, other chronic respiratory diseases, CKD stage 5 and ESRD, CKD stage 1–4, aplastic anaemia, myelodysplastic syndrome, diabetes with chronic complications, diabetes without chronic complications, liver cirrhosis/failure, heart failure, no. of admissions, no. of OPD visits, no. of admissions for ILI, and no. of OPD visits for ILI.

^b^ Hematopoietic stem cell transplant, solid-organ transplantation, immunodeficiency, hematological malignancy, systemic corticosteroids ≥44.1 cDDD, immunosuppressive agents.

^c^ Hematopoietic stem cell transplant, solid-organ transplantation, immunodeficiency and HIV, systemic corticosteroids ≥44.1 cDDD, immunosuppressive agents.

**Table 3.** The association between influenza vaccination and mortality of invasive aspergillosis.

Outcome	Deaths/vaccinated	Deaths/non-vaccinated	cOR (95% CI)	aOR^a^ (95% CI)
60-day mortality after admission	128/3,136,477	208/5,407,974	1.06 (0.85–1.32)	0.82 (0.65–1.04)
90-day mortality after admission	148/3,136,477	250/5,407,974	1.02 (0.83–1.25)	0.83 (0.67–1.03)

Abbreviations: cOR, crude odds ratio; aOR, adjusted odds ratio.

^a^ Adjusting for age, sex, insurance amount, region, systemic corticosteroids, inhaled corticosteroids, immunosuppressive agents, hematopoietic stem cell transplant, solid-organ transplantation, hematological malignancy, metastatic malignancy, solid-organ malignancy, autoimmune diseases, immunodeficiency disorders, HIV, asthma, COPD, other chronic respiratory diseases, CKD stage 5 and ESRD, CKD stage 1–4, aplastic anaemia, myelodysplastic syndrome, diabetes with chronic complications, diabetes without chronic complications, liver cirrhosis or failure, heart failure, no. of admissions, no. of OPD visits, no. for admissions for ILI and no. of OPD visits for ILI.

### Subgroup analyses

In people aged ≥65 years and aged <65 years, vaccination was associated with a lower risk of IA than those without influenza vaccination (aOR [95% CI], 0.85 [0.73–0.99], 0.63 [0.50–0.79], respectively). Only vaccinated males had a reduced risk of IA (aOR 0.78, 95% CI 0.67–0.92). Regardless of people with or without EORTC/MSGERC host factors, vaccination was associated with a lower risk of IA (aOR [95% CI], 0.78 [0.63–0.97], 0.79 [0.67–0.92], respectively). Among people having, immunocompromised status, malignancy, and diabetes, vaccination was significantly associated with a decreased risk of IA (aOR [95% CI], 0.76 [0.61–0.95], 0.80 [0.64–0.99], 0.76 [0.63–0.93], respectively). Among the three influenza seasons, vaccinated people had a significantly lower risk of IA (aOR 0.72, 95% CI 0.59–0.87) only in the 2018–2019 season.

### Sensitivity analyses

Table 4 presents the results of the sensitivity analysis. After changing the IA definition, vaccination was associated with a decreased risk of IA with the use of antifungal agents, pulmonary aspergillosis, and influenza-associated aspergillosis (aOR [95% CI], 0.81 [0.70–0.94], 0.72 [0.61–0.85], 0.52 [0.30–0.90]). This association remained the same (aOR 0.77, 95% CI 0.68–0.88) when we shortened the follow-up time. Vaccinated patients had a reduced risk of hospitalization for influenza and ILI (aOR [95% CI], 0.86 [0.84–0.88], 0.87 [0.87–0.88]). Vaccination was significantly associated with a low risk of fracture when adjusting for all covariates (aOR 0.94, 95% CI, 0.93–0.94), whereas vaccination was not associated with fracture when adjusting for age, sex, region, and insurance amounts (aOR 1.00, 95% CI 0.99–1.01). Hospitalization with influenza was associated with a high risk of IA (aOR 8.54, 95% CI 6.64–10.99, Table 5).

**Table 4.** Sensitivity analyses for the risks of invasive aspergillosis.

	Events/vaccinated	Events/non-vaccinated	cOR (95% CI)	aOR^a^ (95% CI)
Changing IA definition				
IA with using antimycotic agents	324/3,136,469	611/5,407,950	0.91 (0.80–1.05)	0.81 (0.70–0.94)
Any pulmonary aspergillosis	227/3,136,347	469/5,407,756	0.84 (0.71–0.98)	0.72 (0.61–0.85)
Invasive pulmonary aspergillosis	84/3,136,226	169/5,407,502	0.86 (0.66–1.11)	0.81 (0.61–1.07)
Influenza-associated aspergillosis	20/3,136,160	48/5,407,386	0.72 (0.43–1.21)	0.52 (0.30–0.90)
Changing the end of follow-up				
30-day after influenza season	388/3,137,728	739/5,409,913	0.91 (0.80–1.02)	0.77 (0.68–0.88)
Positive control outcome				
Hospitalization with influenza	12,176/3,136,161	17,028/5,407,974	1.23 (1.21–1.26)	0.86 (0.84–0.88)
Hospitalization with ILI	191,269/3,128,774	240,164/5,407,974	1.40 (1.39–1.41)	0.87 (0.87–0.88)
Negative control outcome				
Fracture	87,633/2,603,906	125,803/4,698,975	1.27 (1.26–1.28)	0.94 (0.93–0.94)^b^

Abbreviations: cOR, crude odds ratio; aOR, adjusted odds ratio; IA, invasive aspergillosis; ILI, influenza-like illness.

^a^ Adjusting for age, sex, insurance amount, region, systemic corticosteroids, inhaled corticosteroids, immunosuppressive agents, hematopoietic stem cell transplant, solid-organ transplantation, hematological malignancy, metastatic malignancy, solid-organ malignancy, autoimmune diseases, immunodeficiency disorders, HIV, asthma, COPD, other chronic respiratory diseases, CKD stage 5 and ESRD, CKD stage 1–4, aplastic anaemia, myelodysplastic syndrome, diabetes with chronic complications, diabetes without chronic complications, liver cirrhosis/failure, heart failure, no. of admissions, no. of OPD visits, no. of admissions for ILI, and no. of OPD visits for ILI.

^b^ aOR (95% CI) was 1.00 (0.99–1.01) when the model was only adjusted for age, sex, insurance amount, and region.

**Table 5.** The association between influenza hospitalization and invasive aspergillosis.

	IA/influenza	IA/no influenza	cOR (95% CI)	aOR^a^ (95% CI)
Overall	68/30,181	1111/8,514,270	17.30 (13.54–22.11)	8.54 (6.64–10.99)

Abbreviations: cOR, crude odds ratio; aOR, adjusted odds ratio.

^a^ Adjusting for age, sex, insurance amount, region, systemic corticosteroids, inhaled corticosteroids, immunosuppressive agents, hematopoietic stem cell transplant, solid-organ transplantation, hematological malignancy, metastatic malignancy, solid-organ malignancy, autoimmune diseases, immunodeficiency disorders, HIV, asthma, COPD, other chronic respiratory diseases, CKD stage 5 and ESRD, CKD stage 1–4, aplastic anaemia, myelodysplastic syndrome, diabetes with chronic complications, diabetes without chronic complications, liver cirrhosis or failure, heart failure, no. of admissions, no. of OPD visits, no. for admissions for ILI and no. of OPD visits for ILI.

## Discussion

To our knowledge, this is the first population-based cohort study demonstrating that the influenza vaccination was associated with a reduced risk of IA in the high-risk population. Even in classic high-risk individuals, including those having immunocompromised status and those having EORTC/MSGERC host factors, vaccinated individuals had a lower risk of IA, the same for males and those with malignancy or diabetes.

Influenza vaccination can prevent infection or attenuate the severity of illness, such as outpatient illness, intensive care unit (ICU) admission, pneumonia, and mortality [21]. Pre-existing antibodies and recall of anamnestic immune responses may diminish lung damage and complications [21]. Some studies have indicated that influenza vaccination was associated with a risk reduction of influenza complications. A study from New Zealand suggested that influenza vaccine effectiveness (VE) against ICU admission was higher by reducing the risk of severe complications [22]. Some studies noted that influenza vaccination was associated with a reduced risk of major adverse cardiovascular events, cardiovascular mortality, respiratory diseases, and all-cause mortality [10–12,23]. These studies, along with our results, support the potential benefits of influenza vaccination in reducing the risk of influenza complications.

Our results demonstrate that the association between vaccination and IA varies among the three seasons, and this may be explained by different VE and circulating virus strains in each season as well as by the awareness of influenza-associated aspergillosis. IA was reported to have a high incidence in patients diagnosed with severe influenza and was considered an influenza superinfection after the 2009 H1N1 pandemic [24,25]. Evidence that indicated influenza would increase the risk for IA was published in recent years [26]. Our study also showed more IA cases in 2018–2019 than in the other two seasons. This suggests that the awareness of influenza-associated aspergillosis increased with time. Second, IA may have a higher association with influenza A(H1N1) than with other subtypes. A single-centre study from Canada showed that seven patients were infected with influenza A(H1N1) among the eight influenza-associated aspergillosis cases [6]. Other studies indicated that the circulation of influenza A(H1N1) was correlated with a higher rate of IA [27,28]. Another reason may be that the circulating influenza B lineage was mismatched with the 2017–2018 TIV. The main circulation lineage, influenza B Yamagata lineage, was only included in the 2017–2018 quadrivalent inactive vaccine; as a result, the 2017–2018 TIV was not effective against influenza B [29]. The fourth reason could be the lower VE against influenza A(H3N2), which was the primary circulating strain in the 2016–2017 season [30]. As the circulating influenza strains from the 2016–2017 to 2018–2019 seasons in Taiwan were influenza A(H3N2), influenza B Yamagata lineage, and influenza A(H1N1) and A(H3N2), respectively, the VE may be stronger in the 2018–2019 season. All of these reasons may partially explain the stronger association between vaccination and IA in the 2018–2019 season.

Subgroup analyses showed that vaccination was significantly associated with a reduced risk of IA in most comorbidity groups, except for those diagnosed with chronic respiratory diseases defined in this study. This may be explained by the VE of individuals having chronic respiratory diseases and by other uncontrolled confounders. One study focusing on people at high risk for influenza complications reported that the VE of people with COPD and other respiratory diseases was not effective and that the VE of asthmatic people was lower than that of people who were not at high-risk [31]. In contrast, other studies focusing on COPD patients revealed a significant VE against influenza-associated hospitalization [32,33]. In addition, uncontrolled confounders, including the severity of COPD, use of antibiotics, and airway colonization of *Aspergillus* spp. could affect the results of people diagnosed with chronic respiratory diseases [34–36].

We performed various sensitivity analyses to assess the robustness of our findings. As expected, the results of the analyses using different IA definitions and shortening the follow-up time were similar to the main results. The finding that vaccinated people had a lower risk of hospitalization for influenza than those without vaccination and that people hospitalized with influenza had a higher risk of IA met our hypothesis that vaccination may reduce the risk of IA by preventing influenza and reducing its severity. Sensitivity analysis using the negative control outcome showed an unexpectedly significant association between vaccination and fracture after adjustment for all covariates. As an ideal negative control outcome should be associated with measured and unmeasured confounders and not be associated with exposure, fracture did not meet these conditions [37]. Comorbidities, medications, and health care usage in this study were not confounders for fractures, hence, controlling all the covariates in the model could cause overadjustment. This might explain why there was no significant association between vaccination and fractures when we only controlled for demographics in the model.

This study has some limitations. Although the validity of using the NHIRD to approach influenza vaccination records was unknown, we used the chargeable code and NHI Drug Codes to define vaccination status and ensured that all the people enrolled were qualified to claim this reimbursement, minimizing the probability of misclassification. The study could not detect whether the people had paid for influenza vaccines they received. Second, since IA is difficult to determine and is likely to be underdiagnosed in clinical practice, it added more uncertainty when we used ICD codes to define it. Although we approached several ways to define IA, this information bias could not be avoided or estimated. The NHIRD lacks some sociodemographic factors and health behaviours; thus, potential confounders, including smoking, alcoholism, drug abuse, and malnutrition, could not be controlled for in our results and might have influenced the results. Tables 4 and 5 show the results of sensitivity analyses that support our assumption. However, this was a retrospective observational study and lacked the influenza testing data and the validity of influenza hospitalization defined by ICD codes. We cannot exclude the possibility that these results were affected by measurement error, and our assumption and the influence of influenza strains could not be evaluated thoroughly. According to our assumption, it is better to conduct the analyses using the definition of IAA. As there was more uncertainty in defining influenza using ICD codes and the validity of IAA is unknown, we used IA patients in the study where IA cases included those with IAA. The association between influenza vaccination and IA would be attenuated when the definition of IA is used rather than that of IAA; the results tended to be conservative. Further research is needed to avoid such bias and clarify this causal inference. Finally, we did not find a significant association between influenza vaccination and IA-associated mortality. It could be due to the lower event number compared to IA and the lower measurement accuracy of IA-associated mortality defined by ICD codes and death date after admission.

In conclusion, influenza vaccination was significantly associated with a reduced risk of IA in high-risk individuals; as a result, vaccination may be a possible way to reduce the risk of IA. Our study highlights the insufficient vaccine coverage rate and the need for vaccination in these patients. We suggest that healthcare providers should use these results and encourage them to get vaccinated.

## Supplementary Material

Supplemental Material

## Data Availability

The data that support the findings of this study are available from the Health and Welfare Data Science Center, Ministry of Health and Welfare, Taiwan; however, restrictions apply to the availability of these data, which were under approval for the current study, and therefore are not publicly available. The dataset used in this study was analysed in person at the Health and Welfare Data Science Center, Ministry of Health and Welfare, Taiwan.
