# Supplementary material for: Influenza vaccination is associated with a reduced risk of invasive aspergillosis in high-risk individuals in Taiwan: a population-based cohort study
Source: Emerg Microbes Infect. 2022 Dec 27;12(1):2155584. doi: 10.1080/22221751.2022.2155584 (PMC9809410; doi:10.1080/22221751.2022.2155584)
Supplement: Supplemental Material [file TEMI_A_2155584_SM8630.docx]

**Supplementary Data**

**Methods**

Definition of the catastrophic illness:

To reduce the financial burden for patients with major diseases or injuries, Taiwan’s Ministry of Health and Welfare recommended that patients certified for catastrophic illness are exempted from the co-payment of the National Health Insurance [1]. Catastrophic illness includes malignant neoplasm requiring active or long-term treatment, hereditary coagulation factor deficiency, severe hemolytic anemia, aplastic anemia, chronic renal failure requiring regular dialysis therapy, systemic autoimmune syndrome requiring lifelong treatment, chronic mental disorders, congenital disorders, severe burns, transplantation, long-term mechanical ventilation, disability of at least a moderate degree, major spinal cord injury or pathology, and rare types of diseases [1]

Sensitivity analyses:

There were five types of sensitivity analyses in this study: changing the IA definition, shortening the follow-up time, using positive control outcomes, using negative control outcomes, and accessing the association between hospitalized with influenza and IA. IA definition was converted into four ways: (1) those who were the first hospitalized with aspergillosis and were administered any systemic antifungal agent, (2) those who were the first hospitalized with pulmonary aspergillosis (ICD-10 B44.0 and B44.1), (3) those who were the first hospitalized with invasive pulmonary aspergillosis (ICD-10 B44.0), and (4) those who were first hospitalized with aspergillosis and influenza. The follow-up time was from the start of a season till 30-day after the season ended or till the date before the next season’s public vaccination program; thereafter, vaccination status and IA were followed accordingly. As the definitions of IA and follow-up were changed, we used the new definition to operate the process of enrollment and analysis, respectively. The positive control outcomes were defined as the first hospitalization with ILI or influenza during an influenza season; we excluded people from the original population who were diagnosed within 14 days after vaccination, to perform the analysis. The negative control outcome was defined as whether a person was diagnosed with a fracture from an OPD visit or a hospitalization during the follow-up and never had a fracture before the influenza season (Supplementary Table 2). We excluded people from the original study population who had a fracture before a season and who were diagnosed within 14 days after vaccination, to perform the analysis. Finally, we tested whether people hospitalized with influenza had a higher risk of IA. We defined influenza-associated aspergillosis when a diagnosis of influenza and a diagnosis of aspergillosis were within the same hospitalization. For people who were the first hospitalized with aspergillosis, we assessed whether they were diagnosed with influenza within the same hospitalization. For people who were not diagnosed with IA, we evaluated whether they were hospitalized with influenza during the follow-up period. Then, we analyzed the association between hospitalization with influenza and IA.

Reference

1. Laws & Regulations Database of The Republic of China (Taiwan). Regulations Governing the Exemption of the National Health Insurance Beneficiaries from the Co-Payment 2019 [cited 2022 Oct. 27]. Available from: <https://law.moj.gov.tw/ENG/LawClass/LawAll.aspx?pcode=L0060015>

Supplementary Table 1. ICD codes for defining high-risk comorbidities for influenza complications

| Influenza season | ICD codes |
| --- | --- |
| 2016-17 and 2017-18 | ICD-9-CM: 040-044, 250, 278, 390-398, 402-404, 410-417, 430-438, 440-444, 446, 490-496, 500-508, 510, 514-519, 571, 580-588.  ICD-10-CM: A48, A49, B20, B44, B95, B96, E08-E11, E13, E65-E68, G45-G46, I00-I02, I05-I09, I11-I13, I20-I22, I24-I28, I60-I63, I65-I74, I77, I79, J40-J45, J47, J60-J70, J80-J82, J84, J86, J96, J98, J99, K70, K73-K76, K90, M30-M31, M60, N00-N08, N14-N19, N25, N26, R16, R91. |
| 2018-19 | ICD-10-CM: B18-20, D80-D84, D86, D89, E08-E13, E66, E85, G09, G20, G30-G32, G35-G37, G40, G45-G46, G65, G70, G72, I00-I02, I05-I09, I11-I13, I20-I22, I24-I25, I27-I28, I34-I37, I42-I45, I47-I51, I60-I63, I67-I74, I77, I79, J40-J45, J47, J60-J70, J82, J84, J96, J98, J99, K70-K76, M05-M06, M30-M35, M94.1, N00, N01, N03-N05, N18-N19, N26-N27, Q89.1, Z21, Z90.81. |

Supplementary Table 2. ICD codes for defining comorbidities

| Comorbidities | ICD codes |
| --- | --- |
| Stem cell transplantation | ICD-10-CM: Z94.81, Z94.84, T86.0, T86.5. |
| Solid-organ transplantation | ICD-10-CM: Z94.0, Z94.1, Z94.2, Z94.4, T86.1, T86.2, T86.3, T86.4, Z94.83, Z94.82, T86.81, T86.89, T86.85. |
| Immunodeficiency disorders and HIV | ICD-10-CM for immunodeficiency disorders: D71, D80, D81, D82, D83, D84. |
|  | ICD-10-CM for HIV: B20, Z21. |
| Hematological malignancy | ICD-10-CM: C81, C82, C83, C84, C85, C86, C88, C90, C91, C92, C93, C94, C95, C96. |
| Metastatic malignancy | ICD-10-CM:C77, C78, C79, C80.0, C80.2. |
| Solid-organ malignancy | ICD-10-CM: C00, C01, C02, C03, C04, C05, C06, C07, C08, C09, C10, C11, C12, C13, C14, C15, C16, C17, C18, C19, C20, C21, C22, C23, C24, C25, C26, C30, C31, C32, C33, C34, C37, C38, C39, C40, C41, C43, C44, C45, C46, C47, C48, C49, C50, C51, C52, C53, C54, C55, C56, C57, C58, C60, C61, C62, C63, C64, C65, C66, C67, C68, C69, C70, C71, C72, C73, C74, C75, C76, C80, C4A, C7A. Metastasis malignancy was excluded. |
| Autoimmune diseases | ICD-10-CM: M05, M06, M08, M30, M31, M32, M33, M34, M35, L10, K50, K51, G35, G70.0, M94.1, M36.0, I73.1, D59.0, D59.1. |
| CKD stage 5 and ESRD | ICD-10-CM: I12.0, I13.2, N18.5, N18.6, I13.11. |
| CKD stage 1-4 | ICD-10-CM: I12, I13, N18.  CKD stage 5 and ESRD were excluded. |
| Asthma | ICD-10-CM: J45. |
| COPD | ICD-10-CM: J41, J42, J43, J44. |
| Other chronic respiratory diseases | ICD-10-CM for cystic fibrosis: E84. |
|  | ICD-10-CM for mycobacterial infection: A15, A31.0. |
|  | ICD-10-CM for pneumocystis pneumonia: B59. |
|  | ICD-10-CM for bronchiectasis: J47. |
|  | ICD-10-CM for pneumoconiosis: J60, J61, J62, J63, J64, J65. |
|  | ICD-10-CM for sarcoidosis: D86. |
|  | ICD-10-CM for respiratory failure: J96, Z99.11. |
|  | ICD-10-CM for other pulmonary fibrosis: J84.1. |
| Aplastic anemia | ICD-10-CM: D60, D61. |
| Myelodysplastic syndrome | ICD-10-CM: D46. |
| Diabetes with chronic complications | ICD-10-CM: E08.2, E08.3, E08.4, E08.5, E09.2, E09.3, E09.4, E09.5, E10.2, E10.3, E10.4, E10.5, E11.2, E11.3, E11.4, E11.5, E13.2, E13.3, E13.4, E13.5. |
| Diabetes without chronic complications | ICD-10-CM: E08, E09, E10, E11, E13.  Diabetes with chronic complications was excluded. |
| Liver cirrhosis/failure | ICD-10-CM: K70.2, K70.3, K70.4, K71.7, K72, K74. |
| Heart failure | ICD-10-CM: I11.0, I13.0, I13.2, I50. |
| Influenza-like illness | ICD-10-CM: A15, A20, A22, A24, A36, A37, A70, A78, B34, B39, B59, B97, J00, J02-J06, J09-J18, J20, J21, J40-J42, J44, J69, J80, J85, J90, J92, J94, R05, A21.2, A48.1, A74.8, A74.9, B01.2, B05.2, B25.0, B33.0, B33.4, B33.8, B37.1, B38.0, B38.9, B58.3, J81.0, J86.9, J91.8, J96.0, J96.2, J96.9, J98.1, J98.4, J98.5, R07.0, R06.9, R06.0, R06.2, R06.3, R06.1, R07.1, R09.1, R89.5, R91.1, A02.22, J98.09, R06.82, R06.83, R06.89, R07.81, Z20.810. |
| Influenza | ICD-10-CM: J09, J10, J11. |
| Fracture | ICD-9-CM: 800-827. ICD-10-CM: S02, S12, S22, S32, S42, S52, S62, S72, S82, S92. |

Abbreviations: CKD, chronic kidney disease; COPD, chronic obstructive pulmonary disease; ESRD, end-stage renal disease; HIV, human immunodeficiency virus; ICD, International Classification of Diseases; ILI, influenza-like illness; OPD, Outpatients Department.

Supplementary Table 3. ICD codes for defining medications

| Medication | Codes |
| --- | --- |
| Influenza vaccination | NHI Drug code: J000113277, K000453265, K000453277, K000523206, K000523265, K000492206, K000706206, K000889206, J000113277, X000092206, X000090238, X000090221, X000091221, J000113265, K000901206, K000706277. Code for claiming influenza vaccine injection fee: A2001C |
| Systemic corticosteroids | ATC code: H02AB, H02B |
| Inhaled corticosteroids | ATC code: R03BA, R03AK06, R03AK07, R03AK08, R03AK09, R03AK10, R03AK11, R03AK12, R03AK13, R03AK14, R03AL08, R03AL09, R03AL11, R03AL12 |
| Immunosuppressive agents | ATC code: L01, L04 |
| Antifungal agents | ATC code: J02 |

Abbreviations: ATC code, the Anatomical Therapeutic Chemical code.
